# Editorial: Balancing macronutrients in athletes

**DOI:** 10.3389/fnut.2024.1510423

**Published:** 2024-10-25

**Authors:** Raúl López-Grueso, José Joaquín Muros

**Affiliations:** ^1^Department of Education and Specific Didactics, University Jaume I, Castellón, Spain; ^2^Faculty of Health Sciences, Universidad Isabel I, Burgos, Spain; ^3^Department of Didactics of Corporal Expression, Faculty of Education, University of Granada, Granada, Spain

**Keywords:** athlete, energy, metabolism, performance, nutrition, macronutrient, body composition

The nutritional habits of athletes significantly affect their health and performance throughout a season, as good nutrition allows for adequate body composition and a correct supply of macro and micronutrients, especially when transferred in a practical way to the athlete (1).

Currently, there is a lack of sufficient information on eating patterns, selection factors between diets, nutritional supplementation, and recommendations to meet the needs of athletes (2).

The first study in this Research Topic focused on the dietary intake of semi-professional female football players (Modena et al.). This study examined energy and nutrient intake and Mediterranean diet (MD) adherence in professional football players during a competitive season. Findings point to an energy deficiency in relation to training level, insufficient carbohydrate intake, and risk of insufficient nutrition intake regarding some vitamins and minerals in a substantial percentage of football players. Mean Mediterranean diet adherence indices, however, corresponded with good adherence.

Lak et al., compared the effects of 8 weeks of resistance training combined with two different high-protein diet strategies (immediately pre- and post-exercise and 3 h pre- and post-exercise) in resistance-trained males. Findings reported by this study suggest that a high-protein diet enhances muscular performance and muscle mass in resistance-trained males regardless of timing.

On the other hand (Ryan et al.), focused on uncovering the beliefs and practices of ultra-endurance runners in Ireland regarding carbohydrates in order to gain a better understanding of gastrointestinal symptoms. Findings of this study suggest that gastrointestinal symptoms are prevalent in ultra-endurance athletes. Thus, further research is required to understand the mechanisms behind ultra-endurance-associated gastrointestinal symptoms and to identify best practice for communicating relevant information to the target audience in order to reduce their risk of developing long-term chronic health complications.

Two studies were focused on body composition. Sharn et al., investigated an innovative partnership to integrate training and MUAC (mid-upper arm circumference) *z*-score assessments into a Social Sports Schools program to identify malnutrition risk, demonstrating that this could be used by non-healthcare professionals (non-HCPs) in under-resourced communities and families.

Finally, Lombardo et al., determined the complex interactions between body composition, dietary habits, physical activity, and lifestyle factors, finding differences between genders. Significant gender-specific differences were detected in eating behaviors and food preferences (for example, women felt hungrier in the morning and men in the afternoon), and some behaviors and preferences (taste preferences, nocturnal eating, and uncontrolled eating, among others) varied clearly between FM-to-FFM (fat mass to fat-free mass) tertiles and genders. This ratio (FM-to-FFM) correlated inversely with physical activity levels, especially in general sports engagement and strength training, with the health-related behaviors impacting the BMI and body composition directly.

Thanks to articles like those included in this Research Topic, we have more information about *Balancing macronutrients in athletes*, which promises more practical information for athletes and coaching staff in search of greater performance and better health.
